# Factors associated with pain level in non-cardiac chest pain patients with comorbid panic disorder

**DOI:** 10.1186/s13030-016-0081-5

**Published:** 2016-10-18

**Authors:** Guillaume Foldes-Busque, Stéphanie Hamel, Geneviève Belleville, Richard Fleet, Julien Poitras, Jean-Marc Chauny, Alain Vadeboncoeur, Kim L. Lavoie, André Marchand

**Affiliations:** 1 School of Psychology, Université Laval, 2325, rue des Bibliothèques, Bureau 1116, Québec, G1V 0A6 Canada; 2 Research Centre of the University Affiliated Hospital Hôtel-Dieu de Lévis, 143 rue Wolfe, Lévis, Québec G6V 3Z1 Canada; 3 Research Chair in Emergency Medicine of Laval University, University Affiliated Hospital Hôtel-Dieu de Lévis, 143 rue Wolfe, Lévis, Québec G6V 3Z1 Canada; 4 Department of Family and Emergency Medicine, Université Laval, 1050 Avenue de la Médecine, Bureau 4617, Québec, G1V 0A6 Canada; 5 Research Centre, Montreal Sacré-Coeur Hospital, 5400 Boulevard Gouin Ouest, local K-3000, Montréal, Québec H4J 1C5 Canada; 6 Research Centre, Montreal Heart Institute, 5000 rue Bélanger, Montréal, Québec H1T 1C8 Canada; 7 Psychology Department, Université du Québec à Montréal, C.P. 8888 succursale Centre-ville, Montréal, Québec H3C 3P8 Canada; 8 Fernand-Séguin Research Centre, Louis-Hippolyte Lafontaine Hospital, 7331 rue Hochelaga, Montréal, Québec H1N 3V2 Canada

**Keywords:** Anxiety sensitivity, Cardiac anxiety, Chest pain, Non-cardiac chest pain, Heart-focused anxiety, Panic disorder

## Abstract

**Background:**

Panic disorder (PD) is highly prevalent in patients with non-cardiac chest pain (NCCP). This study aims to explore the role of psychological factors (PD intensity, anxiety sensitivity, heart-related fear, attention and avoidance) common to NCCP and PD in predicting chest pain levels in patients with both conditions.

**Methods:**

This association was investigated in emergency department patients with NCCP and PD receiving either evidence-based treatment of PD or treatment as usual. Patients were assessed at baseline and 14 weeks later for post-treatment.

**Results:**

Only heart-focused fear and attention for cardiac sensations independently explained a significant portion of the variance in baseline pain (*n* = 66). At 3 months follow-up (*n* = 53), changes in heart-related fear was the only factor independently associated with changes in chest pain intensity. Even in patients with PD, fear specific to cardiac sensations seems to play a central role in determining NCCP intensity.

**Conclusion:**

These results suggest that the efficacy of intervention for patients with PD and comorbid NCCP could be improved by targeting heart-related fear and attention.

**Trial registration:**

NCT00736346

## Background

Non-cardiac chest pain (NCCP) is a very common motive for medical consultations [1, 2]. Although medically benign in general, NCCP has major impacts on patients’ functioning and quality of life, limiting daily living activities (e.g., household chores, physical activity and social activity) in as much as 60 % of cases and up to 9 years after initial medical consultation [1, 3–6]. Medical consultations for NCCP cost between eight and 13 billion dollars yearly in the United States [1, 3, 7].

A significant portion of negative outcomes related to NCCP stems from its comorbidity with PD, which is up to 11 times more prevalent in patients with NCCP than in the general population [8–10]. The presence of this anxiety disorder both increases the risk that NCCP will become chronic and the negative impacts on patients’ functioning [11–13].

There are several possible explanations for the strong relationship between PD and NCCP. First, NCCP may occur during a panic attack [10, 13]. However, although first-line interventions for PD remain effective in patients with comorbid NCCP, the effect of such interventions on chest pain appears limited [14]. This could mean that the reduction in PD symptoms, including panic attacks, is not sufficient to decrease the intensity of NCCP. As presented in the major biopsychosocial models, NCCP could rather be a true comorbid condition. Furthermore, these models suggest that the relationship between NCCP and PD may be attributable to common mechanisms and vulnerability factors between both conditions [2, 15]. The most likely psychological mechanisms appear to be anxiety sensitivity (AS) as well as fear, attention, and avoidance of cardiac sensations.

In fact, AS—the belief that anxiety symptoms have negative consequences—seems to be involved in the development and maintenance of NCCP and PD [16, 17]. It has been shown that patients with elevated AS have a lower threshold for pain and an intense fear of pain, both of which naturally result in negative experiences of pain [18, 19].

Heart-focused fear represents catastrophic interpretations of cardiac symptoms [15], which are related with functioning disturbances in NCCP patients and with maintenance of PD [20–22]. Furthermore, fear of pain, a related construct, is strongly associated with excessive attention to pain and the development of related avoidance behaviors [2, 15, 23–25].

Thus, heart-focused attention is also likely involved in the development and maintenance of NCCP [2, 15, 23–25]. This attention to cardiac symptoms contributes to increase the probability of perceiving symptoms and experiencing benign physiological fluctuations as disturbing or painful [23]. In the context of PD and NCCP, heart-focused attention is known to maintain fear of symptoms as well as avoidance of situations and sensations [2, 15, 23–25].

Finally, avoidance is a key factor in the maintenance of PD and NCCP through its impact on fear of symptoms [2, 15, 22, 26]. It can also directly affect pain by promoting physical deconditioning via physical activity avoidance. In turn, deconditioning tends to exacerbate painful physical symptoms, thereby reinforcing fear and attention, which in turn reinforces avoidance [27].

To our knowledge, no study to date has specifically explored the relationships between AS, as well as fear, attention, and avoidance of cardiac sensations in patients with comorbid PD and NCCP. A clearer understanding of these relationships is essential to increase the efficacy of interventions for patients with both conditions.

The first objective of this study is to investigate the relationships between AS, heart-focused fear, attention and avoidance, as well as panic symptoms, and chest pain level in patients with comorbid PD and NCCP. The second objective is to document the relationships between changes in AS, heart-focused fear, attention and avoidance, as well as panic symptoms, and reduction in perceived chest pain intensity following evidence-based intervention for PD or usual care.

## Methods

### Participants and setting

Patients were recruited from three academic emergency departments (EDs) between November 2005 and December 2009 in the course of a larger trial [14]. Patients aged 18 years or older presenting a chief complaint of chest pain deemed to be of non-cardiac origin by the ED physician (i.e., negative serial electrocardiogram and cardiac enzyme tests, and no identifiable cause on chest radiography) were considered for inclusion in the study. The inclusion criteria were as follows: fluent in written and oral English or French and a primary diagnosis of PD. Patients were excluded if they had a history of cardiac disease, had received psychological or psychopharmacological intervention in the past 6 months, or presented a condition that could invalidate the interview (e.g., psychotic state, intoxication, schizophrenia, dementia, cognitive impairment, intellectual deficiency). Only patients who completed all measures at pre-intervention were included in the study while only those who completed post-intervention data were included in the prospective analysis (objective 2).

### Measures

Demographic and medical interview: A structured interview was used to collect sociodemographic data and medical history.

The Anxiety Disorders Interview Schedule for Diagnostic and Statistical Manual of Mental Disorders, Fourth Edition (ADIS-IV) [28]. This instrument was used to diagnose PD. The diagnosis reliability of the ADIS-IV for PD was 92 % in a randomly selected sample including 30 % of the interviews [14].

The Panic and Agoraphobia Scale (PAS) [29, 30]: This 13-item instrument assesses the severity and frequency of PD and agoraphobia symptoms over the past week. It has good internal consistency and satisfactory inter-rater and test-retest reliability [29, 30].

The Anxiety Sensitivity Index (ASI) [31, 32]: This 16-item questionnaire measures the tendency to attribute negative consequences to anxiety symptoms (anxiety sensitivity). This questionnaire has good internal consistency (α = .82 to .91), good temporal stability, and satisfactory test-retest reliability [32].

The Short-form McGill Pain Questionnaire, chest pain version (SF-MPQ-CP) [33]: This 15-item questionnaire was used to measure subjective chest pain intensity. It has good internal consistency and very good test-retest reliability [34].

The Cardiac Anxiety Questionnaire (CAQ) [35]: This 18-item questionnaire measures heart-focused anxiety. Its three subscales assess heart-focused fear, heart-focused attention, and avoidance of cardiac sensations. This questionnaire has good internal consistency (α = 0.83) and satisfactory convergent validity [35, 36]. Each of the three CAQ subscales was analyzed independently.

### Procedure

Graduate students in clinical psychology assessed NCCP patients in each of the three sites. PD and other disorders were evaluated using the ADIS-IV [28]. Eligible patients were subsequently invited to complete the pre-intervention assessment and were assigned to one of four conditions (one session of cognitive-behavioral therapy [CBT], seven sessions of CBT, pharmacological intervention with paroxetine, or treatment as usual as ordered by ED physician) using a randomized cohort design [14]. Intervention conditions (cohorts) were randomized rather than individuals according to a predetermined sequence, allowing each cohort to be recruited during multiple 3-month periods. All patients were invited for assessment 14 weeks after intervention initiation. The detailed procedure has been published elsewhere [14].

### Statistical analyses

Two groups of patients were created. The first group included the entire sample (cross-sectional sample); the second included only patients with post-intervention data (prospective sample). This approach was chosen as 18.5 % of patients presented missing data. Analyses of variance were conducted to compare patients with post-intervention data with patients without post-intervention data on continuous variables. Comparisons on categorical variables were conducted with χ^2^ test or Fisher’s exact test.

For the first objective, a Pearson’s correlation matrix was generated to identify factors significantly related to chest pain in the cross-sectional sample among AS, components of heart-focused anxiety, PD intensity and age. All variables significantly associated with SF-MPQ-CP scores were entered in the first step of a hierarchical linear regression analysis to determine their independent contribution to pain level. As a second step, sex was added to the model, as it has been shown to influence how AS relates to NCCP [37].

For the second objective, change scores were created to establish relationships between changes on the ASI, PAS, CAQ subscales, and SF-MPQ-CP in the prospective sample. These scores were obtained by subtracting post- from pre-intervention results. A correlation matrix was used to identify relationships between change scores on the ASI, PAS, and CAQ subscales, and those on the SF-MPQ-CP as well as age of participants. Variables significantly correlated with change in chest pain intensity were included in the first step of a hierarchical linear regression analysis with change in SF-MPQ-CP scores as the dependant variable. Sex was added as a potential confounding variable in the second step.

## Results

### Participants

**Fig. 1:**
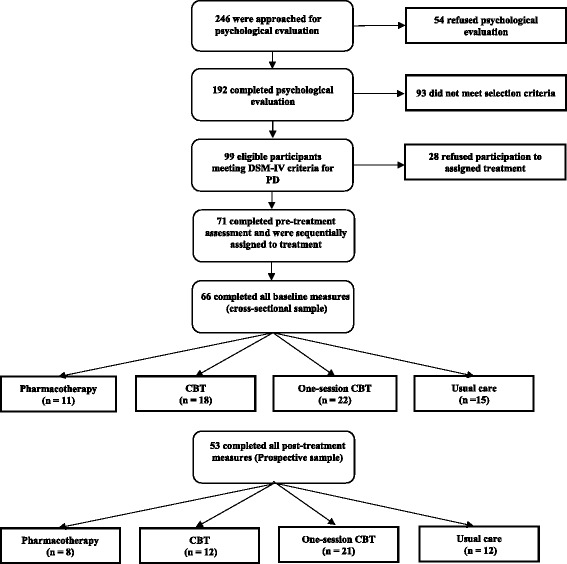
Flowchart of recruitment

A total of 99 eligible patients were identified; 71 agreed to participate in the study. Sixty-six patients completed all baseline measures and were included in the sample. Eighty-one percent (*n* = 53) of participants provided post-intervention data and were included in the prospective group (see Fig. 1). The sample with complete post-intervention data was not significantly different from the sample with incomplete post-intervention data (see Table 1 for a summary of sample characteristics).

**Table 1:** Sample characteristics

Characteristics	Cross-sectional sample (*n* = 66)	Prospective sample (*n* = 53)	Patients with missing post-intervention data (*n* = 13)	p*
Mean age (SD)	41.76 (13.6)	41.45 (13.42)	43.00 (11.85)	.705
Female, n (%)*	31 (47)	22 (42)	9 (69)	.073
Married or common-law, n (%)**	38 (58)	31 (59)	7 (54)	.761
Family income ≥ $60,000, n (%)**	24 (36)	19 (36)	5 (39)	1.00
Education				.343
≥ 12 years, n (%)**	36 (55)	29 (55)	9 (69)	
Currently employed, n (%)	48 (73)	40 (75)	8 (62)	.319
Comorbid psychiatric disorders, n (%)	44 (67)	34 (64)	10 (77)	.522
Anxiety disorders, n (%)	43 (65)	34 (64)	9 (69)	1.00
Mood disorders, n (%)	10 (15)	6 (11)	6 (31)	.103
CAQ – Heart-focused fear subscale				
Baseline, mean (SD)	2.39 (0.75)	2.36 (0.77)	2.49 (0.69)	.578
Mean change score (SD)	-	0.85 (0.75)	-	
CAQ- Avoidance subscale				
Baseline, mean (SD)	1.38 (0.98)	1.34 (0.92)	1.58 (1.24)	.436
Mean change score (SD)	-	0.32 (0.81)	-	
CAQ – Heart-focused attention subscale				
Baseline, mean (SD)	1.87 (0.75)	1.84 (0.78)	1.98 (0.62)	.568
Mean change score (SD)	-	0.46 (0.85)	-	
PAS				
Baseline, mean (SD)	19.62 (9.11)	19.17 (8.48)	21.46 (11.55)	.421
Mean change score (SD)	-	12.23 (8.56)	-	
ASI				
Baseline, mean (SD)	28.98 (10.57)	28.72 (10.69)	30.08 (10.42)	.681
Mean change score (SD)	-	7.81 (11.53)	-	
SF-MPQ-CP				
Baseline, mean (SD)	16.53 (10.99)	17.06 (10.41)	14.38 (13.39)	.437
Mean change score (SD)	-	7.32 (10.98)	-	

*p < 0.05 Prospective sample vs patients with missing post-intervention data

**Data was missing for some patients

*CAQ* cardiac anxiety questionnaire, *PAS* panic and agoraphobia scale, *ASI* anxiety sensitivity index, *SF-MPQ-CP* short-form McGill pain questionnaire, chest pain version

### Relationships between CAQ subscales, AS, panic symptoms, age and pain

Only the CAQ heart-focused attention subscale (*r* = 0.465; *p* < 0.01) and heart-focused fear subscale (*r* = 0.270; *p* < 0.05) scores were significantly correlated with SF-MPQ-CP score (see Table 2). Together, these two variables explained 19.5 % of the variance in SF-MPQ-CP score (*F* (2, 63) = 8.857; *p* < 0.001). Sex did not significantly contribute to the model (results not shown). See Table 3 for further details.

**Table 2:** Correlation matrix for CAQ subscales, ASI, PAS, SF-MPQ-CP and age (*n* = 66)

Variables	1	2	3	4	5	6	7
1. CAQ – Fear Subscale	1						
2. CAQ – Avoidance subscale	.308*	1					
3. CAQ – Attention subscale	.460**	.311*	1				
4. PAS	.264*	.184	.327**	1			
5. ASI	.338**	.126	.431**	.456**	1		
6. SF-MPQ-CP	.270*	.181	.464**	.164	.162	1	
7. Age	.006	.058	.054	.000	-.075	-.194	1

* p < 0.05; ** *p* < 0.01

*CAQ* Cardiac Anxiety Questionnaire, *PAS* Panic and Agoraphobia Scale, *ASI* Anxiety Sensitivity Index, *SF-MPQ-CP Short-form McGill Pain Questionnaire, chest pain version*

**Table 3:** Multiple regression analysis predicting pain scores

	B	(95% CI)	β	sr^2^	Adjusted *R* ^2^	Δ*R* ^*2*^
Baseline scores (*n* = 66)						
CAQ – Fear subscale	1.046	(-2.615- 4.708)	0.072	0.004	0.195	0.219*
CAQ – Attention subscale	6.344	(2.659- 10.029)	0.431	0.147*		
Change scores (*n* = 53)						
CAQ – Fear subscale	5.368	(0.312-10.425)	0.367**	0.070	0.203	0.264**
CAQ – Attention subscale	2.406	(-2.587- 7.399)	0.185	0.014		
CAQ – Avoidance subscale	-0.120	(-4.434- 4.194)	-0.009	0.000		
ASI	0.019	(-0.309- 0.347)	0.020	0.000		

*p ≤ 0.001

**p = 0.005

*CAQ* Cardiac Anxiety Questionnaire*, ASI* Anxiety Sensitivity Index

### Relationships between changes in CAQ subscales, AS, panic symptoms, age and changes in pain at post-intervention

Change on all measures except the PAS were significantly correlated with reduction in SF-MPQ-CP scores (see Table 4). The multivariate model explained 20.3 % of the variance in change in SF-MPQ-CP scores, *F*(4,48) = 4.315; *p* < 0.01. However, only change in the CAQ heart-focused fear subscale contributed significantly to the model (*p* = 0.038) (see Table 3). Including sex in the model did not significantly alter the results (results not presented).

**Table 4:** Correlation matrix for CAQ subscales, ASI, PAS, age and SF-MPQ-CP change scores (*n* = 53)

Change scores	1	2	3	4	5	6	7
1. CAQ – Fear Subscale	1						
2. CAQ – Avoidance subscale	.504**	1					
3. CAQ – Attention subscale	.638**	.595**	1				
4. PAS	.167	.400**	.153	1			
5. ASI	.595**	.453**	.649**	.179	1		
6. SF-MPQ-CP	.493**	.295*	.427**	.215	.355**	1	
7. Age	.100	.059	.184	.128	-.021	.081	1

*p < 0.05; ** *p* < 0.01

*CAQ* Cardiac Anxiety Questionnaire, *PAS* Panic and Agoraphobia Scale, *ASI* Anxiety Sensitivity Index, *SF-MPQ-CP Short-form McGill Pain Questionnaire, chest pain version*

## Discussion

The primary objective of this study was to evaluate the association between AS, heart-focused fear, attention and avoidance of cardiac symptoms, as well as panic symptoms, and perceived chest pain level in patients with PD and NCCP. The association between changes in these psychological factors and reduction in pain scores following evidence-based intervention for PD or usual care was also assessed.

Of the variables evaluated in this study, only heart-focused fear and attention for cardiac sensations (heart-focused attention) were associated with NCCP intensity; together, they explained 19.5 % of the variance in pain scores. However, only the effect of attention was statistically significant.

The prospective analysis revealed that change in AS, as well as in fear, attention, and avoidance of cardiac sensations were correlated with changes in NCCP over a period of 14 weeks of evidence-based intervention for PD or usual care. However, only change in fear contributed significantly to change in chest pain intensity, independently of other factors, explaining 20.3 % of its variance.

At first glance, the absence of significant results for avoidance, PD severity, and AS seems to contradict theoretical models of NCCP, as well as the literature in this area [2, 10, 16]. However, although AS and PD symptoms have been shown to be related to NCCP, the relationships do not necessarily imply that these are relevant in a sub-population of individuals who already present PD and high levels of AS. In fact, the results of the correlation matrix suggest that AS and PD symptoms could indirectly contribute to NCCP through their strong relationship with heart-focused fear and heart-focused avoidance. This hypothesis is consistent with Eifert and colleagues (2000) who proposed that heart-focused anxiety, a construct comprising heart-focused fear, heart-focused attention, and cardio-protective avoidance, is a distinct construct of AS [15]. More specifically, AS may be a predisposing factor for both PD and fear, attention and avoidance of cardiac symptoms [15].

Furthermore, avoidance does not seem to contribute to chest pain independently of its relationship with other dimensions of heart-focused anxiety. There are two main explanations for this finding. First, avoidance is generally conceptualized as a response designed to manage fear of symptoms [2, 15, 38]. This explanation is supported by stronger correlations (*r* = 0.4 to 0.5) between change in avoidance and change in AS, heart-focused attention, and fear of cardiac symptoms than between change in avoidance and change in reported pain (*r* = 0.3). Another possibility is that avoidance contributes to pain-related disability, rather than to pain itself. Furthermore, the absence of an effect of fear independent of the effect of heart-focused attention on perceived pain may be explained by the central role of attention in pain perception [2, 15, 23]. That is, the role of fear may, in this instance, be limited to increasing attention for chest pain. This result is consistent with the literature suggesting that fear of symptoms increases vigilance, thus promoting greater perception and fear of symptoms while maintaining and increasing attention [2, 15, 23].

In the prospective component of the study, only change in heart-focused fear was related to change in pain, independently of change in AS, heart-focused avoidance, and heart-focused attention. This result suggests a central role of fear in the experience of NCCP. Furthermore, the observed inter-correlations indicate that change in fear is the variable most strongly correlated with change in all other variables. This result is also consistent with the literature, and with models of PD, NCCP and common vulnerability factors for pain and anxiety [2, 39, 40].

The apparently contradictory results between the cross-sectional and prospective components of the study can be reconciled by highlighting the importance of heart-focused attention and heart-focused fear in perceived chest pain. Attention plays a central role in pain perception. In fact, leading theoretical models for pain suggest that change in pain is essentially attributable to change in attention to pain [2, 15, 26].

The negative findings concerning the role of AS and PD severity in determining pain severity in this sample could be due to specific characteristics of patients with PD and comorbid NCCP. Studies have shown that PD severity and the level of AS are lower in PD patients with comorbid NCCP than in patients with PD alone [41, 42]. These characteristics are often interpreted as a sign of a more recent onset of PD in ED patients with NCCP than in other patients with PD. This said, one could hypothesize that heart-focused anxiety would be higher in patients with PD and NCCP, but, to our knowledge, no study has investigated this question yet.

Overall, the results of this study suggest that NCCP is a condition independent of PD instead of a symptom of this anxiety disorder. The results of this study also provide a possible explanation for the relatively weak effect of intervention for PD on NCCP severity. Although PD intervention generally reduces AS and fear of symptoms, it appears that directly targeting fear of cardiac symptoms is essential for reducing NCCP intensity. This is not surprising since chest pain is the defining symptom of NCCP. However, none of the exposure exercises used in cognitive-behavioural therapy reliably provoke chest pain. Furthermore, modification of interpretation of symptoms mostly addresses the link between symptoms and anxiety, which may be insufficient to impact fear of cardiac symptoms. In this case, the addition of a module that more specifically targets heart-focused fear and heart-focused attention could increase the efficacy of PD intervention to reduce comorbid NCCP.

The present study has some limitations that must be taken into consideration in interpreting the results. First, some of our negative results may be attributable to the limited sample size, particularly for the prospective component of the study. Second, the present study addressed AS, heart-focused attention, heart-focused fear, cardio-protective avoidance, and PD in the context of NCCP. However, NCCP and PD are also associated with other psychological factors such as alexithymia and neuroticism [16], which may constitute confounding factors. Furthermore, although exploration of correlated factors generates hypotheses for observed inter-relationships, hypotheses can only be confirmed or disconfirmed by more focused research designs.

The primary strengths of this study are the inclusion of both cross-sectional and prospective components, and the use of well validated measures. Finally, to our knowledge, this is the first study to document variables related to NCCP intensity and change in variables related to NCCP levels in a sample of PD patients.

## Conclusions

Heart-focused fear and heart-focused attention explain a significant portion of level of chest pain in PD patients with NCCP. Decrease in fear of sensations seems to be a central factor implicated in NCCP reduction. The efficacy of intervention for patients with PD and NCCP could be improved by more specifically targeting this construct.
